# Opportunities for topical antimicrobial therapy: permeation of canine skin by fusidic acid

**DOI:** 10.1186/s12917-017-1270-6

**Published:** 2017-11-21

**Authors:** Sian-Marie Frosini, Ross Bond, Anette Loeffler, Jo Larner

**Affiliations:** 1 Department of Clinical Sciences and Services, Royal Veterinary College, Hawkshead Lane, North Mymms, Hatfield, UK; 2 Research Centre for Topical Drug Delivery and Toxicology, Department of Pharmacy, Pharmacology & Postgraduate Medicine, University of Hertfordshire, Hatfield, UK

**Keywords:** Canine, Skin, Topical therapy, Pyoderma, Fusidic acid

## Abstract

**Background:**

Staphylococcal infection of the canine epidermis and hair follicle is amongst the commonest reasons for antimicrobial prescribing in small animal veterinary practice. Topical therapy with fusidic acid (FA) is an attractive alternative to systemic therapy based on low minimum inhibitory concentrations (MICs, commonly <0.03 mg/l) documented in canine pathogenic staphylococci, including strains of MRSA and MRSP (methicillin-resistant *Staphylococcus aureus* and *S. pseudintermedius*). However, permeation of canine skin by FA has not been evaluated in detail. This study aimed to define the degree and extent of FA permeation in canine skin in vitro from two sites with different hair follicle density following application of a licensed ophthalmic formulation that shares the same vehicle as an FA-betamethasone combination product approved for dermal application in dogs. Topical FA application was modelled using skin held in Franz-type diffusion cells. Concentrations of FA in surface swabs, receptor fluid, and transverse skin sections of defined anatomical depth were determined using high-performance liquid chromatography and ultraviolet (HPLC-UV) analysis.

**Results:**

The majority of FA was recovered by surface swabs after 24 h, as expected (mean ± SEM: 76.0 ± 17.0%). FA was detected within 424/470 (90%) groups of serial sections of transversely cryotomed skin containing follicular infundibula, but never in 48/48 (100%) groups of sections containing only deeper follicular structures, nor in receptor fluid, suggesting that FA does not permeate beyond the infundibulum. The FA concentration (mean ± SEM) in the most superficial 240 μm of skin was 2000 ± 815 μg/g.

**Conclusions:**

Topically applied FA can greatly exceed MICs for canine pathogenic staphylococci at the most common sites of infection. Topical FA therapy should now be evaluated using available formulations in vivo as an alternative to systemic therapy for canine superficial bacterial folliculitis.

**Electronic supplementary material:**

The online version of this article (10.1186/s12917-017-1270-6) contains supplementary material, which is available to authorized users.

## Background

Antibiotic resistance is a major threat to global health and modern medicine [1]. Canine pyoderma caused by *Staphylococcus pseudintermedius* is amongst the commonest reasons for prescribing antimicrobial drugs in small animal veterinary practice [2]. Traditionally in canine practice, surface infections (confined to the inter-follicular epidermis) are treated topically, whereas superficial infections such as bacterial folliculitis (that extend to the follicular infundibulum without extension into the surrounding dermis) are treated with oral antibiotics. The recent emergence of methicillin-resistant *S. pseudintermedius* (MRSP) [3] that are routinely resistant to licensed oral antibiotics has renewed interest in the direct application of topical antibiotics and antiseptics for superficial pyoderma [4, 5].

Fusidic acid (FA) is an antibiotic which has a steroid-like structure, with proven activity in vitro against coagulase-positive staphylococci including MRSP [6, 7]. The physico-chemical properties of this large, lipophilic molecule (molecular weight of 517 kDa, octanol/water partition co-efficient >4, 6 free hydrogen bonding groups) predict limited diffusivity through stratum corneum and restricted partitioning to the more hydrophilic living epidermis, after topical application [8, 9]. These features correlate with clinical efficacy of licensed FA-containing topical veterinary products in surface infections such as canine acute moist [pyotraumatic] dermatitis [10]. Utility in canine superficial pyoderma, however, is dependent upon adequate permeation into hair follicles, but this has received little attention. Studies of clinical efficacy of topical FA in canine superficial pyoderma / bacterial folliculitis are lacking [4, 11].

Stuttgen and Bauer established that in sparsely-haired human skin, FA is limited to the stratum corneum and epidermis after topical gel application, and does not penetrate into the deep dermis or subcutaneous fat [12]. By contrast, Degim et al. reported that 1.3% of FA in a betamethasone-containing gel formulation penetrated full-thickness haired canine skin in diffusion cell studies [13]. Skin integrity was not assessed prior to gel application, and FA was quantified in only receptor fluid and not on or within skin itself [13].

In view of these prior conflicting and incomplete data, we developed an in vitro model of topical FA application using canine skin held in Franz-type diffusion cells and high-performance liquid chromatography and ultraviolet (HPLC-UV) analysis of FA concentrations to define the degree and extent of drug permeation in skin from sites with varying hair follicle density. We describe for the first time how the depth of drug permeation into dermal layers can be defined by concurrent observation, in representative paired transverse histological sections [14, 15], of the variations in hair follicle anatomy that mark the infundibulum, isthmus and inferior portions of hair follicles. In addition, conventional analyses of drug recovery in receptor fluid and swabs from surface of dosed skin complemented evaluation of dermal drug concentrations. These data were used to inform likely clinical utility in canine superficial and deep pyoderma.

## Methods

### HPLC-UV detection of fusidic acid.

#### Validation

Fusidic acid sodium salt (≥98%, Sigma-Aldrich, Irvine, UK) was diluted in absolute ethanol to produce standard (0.5–49 μg/ml) and quality control (0.5, 1.0, 6.5 and 40 μg/ml) solutions (see Additional file 1 for chemicals used). High Performance Liquid Chromatography – ultraviolet analysis (HPLC-UV) was performed using an Ultimate 3000 (Thermo Scientific, Paisley, UK) system comprising quaternary pump, autosampler, column oven and diode array detector. The column was from Kinetex (C18 2.1 mm × 50 mm, 1.7 μm particle size; Phenomenex, Macclesfield, UK) held at 35 °C. Mobile phase A comprised methanol; mobile phase B 0.1 M acetic acid. Mobile phase A/B was ramped from 30/70 to 78/22 *v*/v ratio over 4 min and then held for 5 min. Mobile phase A/B then returned to 30/70 over 30 s and re-equilibrated for 7 min. The flow rate was maintained at 0.35 ml/min. The retention time of FA was 9.1 min, with UV detection at 240 nm.

Samples were analysed using a validated method developed at the University of Hertfordshire in accordance with OECD guidelines for studies of skin absorption in vitro [16]. Injection volume was 2 μl. The linear dynamic range for FA, based on peak areas with 1/x^2^ weighted regression was 1.24–249 ng on column (*R* ^2^ = 0.9998), with limit of detection (LOD) of 0.50 ng on column (signal/noise = 3). System precision, determined using replicate injections (*n* = 10) at 1.24 and 187 ng on column, was 6.6 and 0.76%, respectively. Receptor fluid, cotton wool swab and canine cryosection extracts (from 8 different animals) demonstrated no matrix interference at the retention time of FA. The calibration range was 0.5–49 μg/ml. Satisfactory intra and inter-run accuracy (87–107%) and precision (±15%) was obtained at low (1.0 μg/ml), mid (6.5 μg/ml) and high (40 μg/ml) concentrations of the calibration curve and at the limit of quantification (LOQ, 0.5 μg/ml). Extraction efficacies of FA from spiked canine skin cryosections (at low, mid, and high QC levels, 8 replicates of each) using ethanol confirmed recoveries to be 98.7–101.3%. Stabilities of spiking solutions and spiked matrices were shown to be at least 2 weeks when refrigerated. All experimental samples were analysed within 14 days of refrigerated storage.

#### Analysis of samples

Standard solutions covering the calibration range were run at the start of each batch with low, mid and high QCs bracketing no more than 15 test samples. Batch sample data were accepted when the accuracy and precision of these met validation criteria. In order to obtain concentrations of FA found in skin samples, the amount of FA found in the skin was adjusted for the sample weight from which the sample was obtained.

### Canine skin collection

Full thickness canine skin was obtained from healthy Beagle dogs (three male, three female, aged 6–12 months, 8–14 kg) immediately after euthanasia for reasons unrelated to this study (approved by the Royal Veterinary College’s Clinical Research Ethical Review Board 2016 1651–2-R). Hair was clipped (Moser Arco 1854, Wahl, Sterling, IL, USA) to within 3 mm of skin surface taking care not to damage skin integrity, and skin was then excised from the dorsum and groin of each dog, immediately wrapped in tin foil and chilled by frozen ice blocks prior to storage at -20 °C within 6 h of collection. Harvested skin was used within 5 months [17].

### Skin measurements

#### Thickness of whole skin specimens

The thickness of the centre of each 3 × 3 cm portion of skin was measured using callipers immediately before assembly into the diffusion cells, as described below.

#### Thickness of stratum corneum

Vertical cryosections through full thickness skin were prepared from undosed skin from each treatment group (undamaged, shampoo-treated and tape-stripped skin) for both dorsum and groin for all six animals (total *n* = 36). Eight cryosections were taken from each piece of skin, sectioning both from panniculus to epidermis (*n* = 4) and from epidermis to panniculus (*n* = 4), and stained with haematoxylin and eosin. Thickness of stratum corneum was measured at three points per section where stratum corneum was at its most compact / intact and not obviously folded [18] using a light microscope (×40 magnification) and Image-Pro Plus v5.0.1.11 software (Media Cybernetics, Duxford, UK).

#### Hair follicle density

Hair follicle density (compound follicles per mm^2^) and infundibular area (as a percentage of skin area) at the level of the common infundibulum were compared in replicate control untreated dorsal (*n* = 6) and groin (*n* = 6) skin samples by microscopy of transverse haematoxylin and eosin-stained paraffin sections of skin from one male and one female Beagle dog. The hair follicle count and area were measured at three randomly selected areas per section using microscope settings and software as for stratum corneum measurements described above.

#### Electrical resistance

Electrical resistance between saline treated epidermal skin surface and receptor fluid was used to assess skin barrier integrity in each assembled diffusion cell using an ohmmeter (Iso-Tech LCR-821 Meter, Iso-Tech, Southport, UK) [19].

### Diffusion cell experiment

Four dermal absorption experiments with full thickness canine skin were conducted using a 10 mg/g FA suspension (Isathal®, Dechra Veterinary Products (DVP), Shropshire, UK) which contains the same vehicle as a licensed topical skin product for dogs (Isaderm®, DVP). Dorsal and groin skin sourced from six animals (three dogs per experiment) was defrosted and defatted by blunt dissection before division into three evenly sized pieces (70 cm^2^), one for each treatment group and assembled into diffusion cells containing receptor fluid within 3 h. For each experiment, 21 static Franz diffusion cells (Permgear Inc. Hellertown, PA USA) were assembled with portions of either dorsal or groin skin (3 cm × 3 cm) which had either been left untreated (*n* = 6), repeatedly tape-stripped (*n* = 6) to mimic damage to the stratum corneum, or shampooed with a 2% chlorhexidine and 2% miconazole shampoo (Malaseb®, DVP) to mimic clinical use (*n* = 6). One diffusion cell containing untreated skin from each animal was assembled but left undosed (negative controls). Skin allocated to be tape-stripped was quickly [20] and repeatedly (*n* = 30) [20] stripped with D-Squame discs (22 mm; Cuderm, Dallas, TX, USA) prior to assembly into the cells. A uniform pressure was applied to each disc for 2 s using a 225 g/cm^2^ applicator before disc removal with forceps. The epidermal surface of relevant portions of excised skin (approximately 70 cm^2^) were moistened and shampooed (0.02 ml/cm^2^) for 2 min by hand, then left for 10 min, as per label instructions for clinical use, prior to rinsing with water (2 × 5 ml). Treated skin was blotted dry with paper towels and cut into pieces for assembly into the cells.

Each piece of skin was placed between a glass receptor chamber, containing measured volumes (approximately 14 ml) of ethanol / pH 5.0 phosphate buffered saline, 25/75 *v*/v [13], and a magnetic stirrer, and glass donor chamber and secured by pinch clamp, exposing 1.77 cm^2^. Diffusion cells were randomly assigned positions in stirrer blocks and plumbed into a heated water circulator system in order to maintain a constant skin surface temperature of 32.0 ± 1.0 °C confirmed by infrared camera (P620, FLIR, West Malling UK). Skin barrier integrity was established in each cell by measurement of electrical resistance as described above.

After equilibration overnight, 18 of the 21 assembled cells were dosed with 100 μl of the FA suspension using a calibrated positive displacement pipette. Saline (0.9%, 100 μl) was added by pipette to all cells in order to liquefy the gel [13]. A glass rod was used to gently spread the gel across the entire exposed surface of skin. The total amount of FA applied to each cell was determined by weighing the filled and emptied pipette tip and the glass rod before and after use. The donor chambers were then promptly occluded using plastic paraffin film.

Receptor fluid samples (250 μl) were collected from each cell before (pre-dose) and at 12 and 24 h after dosing, with replacement of equal volumes of fresh receptor fluid at each time point. Additional sampling intervals were deemed unnecessary in anticipation of negligible penetration into the receptor fluid. After 24 h, the donor chamber was removed and the surface skin and inside of the donor chamber were both swabbed with cotton wool. Swabs were transferred to glass vials and soaked in ethanol (10 ml) for a minimum of 24 h at 4 °C to extract the FA. Aliquots of the swab extracts and receptor fluid samples were transferred to autosampler vials (2 ml) and crimped capped. Skin specimens were gently removed from the receptor chamber using forceps, taking care not to touch the exposed area of skin, and were stored in foil at −70 °C prior to cryosectioning (to minimise drug lability in the skin).

FA concentrations in ethanolic swab extracts and receptor fluid samples were determined by direct injection of aliquots, transferred to autosampler vials, using the HPLC-UV method described above.

### Skin cryosectioning

**Fig. 1:**
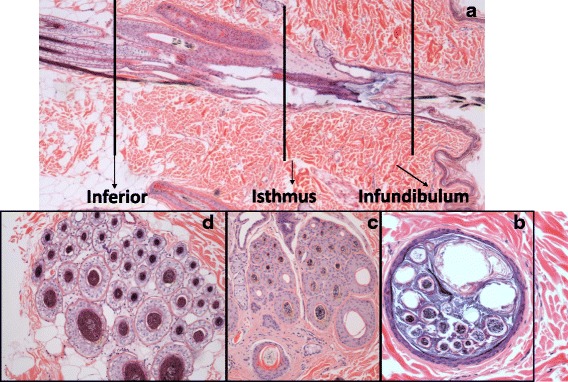
Composite image of the histology of the canine compound hair follicle. **a** Traditional vertical section through the long axis of a compound follicle from epidermis (right) to panniculus adiposus (left). Lines indicate planes of section for corresponding transverse images that define depth of section. **b** Transverse section at common infundibulum: follicle is lined by stratified squamous keratinising epithelium that recapitulates that of the interfollicular epidermis and contains multiple naked hair fibres. **c** Transverse section at isthmus: compound follicle comprises a cranial primary hair and a group of (commonly 14–18) secondary hair follicles; each hair shaft is surrounded by root sheaths whose anatomy varies with stage of hair growth. **d** Transverse section at inferior portion of follicles: presence indicates anagen phase represented by hair fibre surrounded by inner root sheath and glycogen-rich outer root sheath

OCT-embedded frozen specimens of skin were cryosectioned transversely starting from the deep dermis proceeding towards epidermis in groups of seven sections, to avoid cross contamination of sections with the blade. Each group comprised a) five sequential 20 μm sections that were placed in individual glass vials and extracted in ethanol (5 ml) for 24 h, for subsequent FA HPLC-analysis, and b) a further two 10 μm sections which were mounted on Polysine™ slides (ThermoFisher Scientific, Paisley, UK) prior to staining with haematoxylin and eosin. The stained slides were examined by light microscopical observation of hair follicle anatomy and presence of other skin structures by a blinded assessor (RB) to determine the anatomical depth within the skin (Figure 1). As the highest proportion of hair follicle infundibula were present in the uppermost two vials (equivalent to a depth of approximately 240 μm) these were selected for determination of the maximum FA concentration achievable in the superficial skin layers in these experiments.

### Statistical analyses

Statistical analyses utilised IBM SPSS Statistics Package 21 (IBM, Portsmouth, UK) with *P* values of ≤0.05 considered significant. Normality was assessed through Shapiro-Wilk test prior to use of either the Kruskal-Wallis test with Dunn’s *post-hoc* test, or one-way ANOVA with Bonferroni *post-hoc* correction as appropriate. Chi-squared tests evaluate contingency table data.

## Results

### FA recovery

**Fig. 2:**
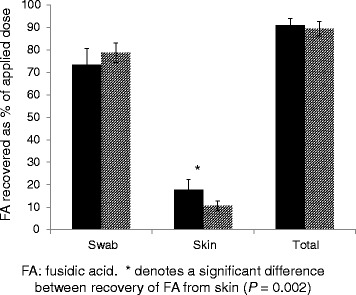
Individual / combined percentage recoveries of FA (mean ± SEM) in skin and swab following 24 h topical application of a 10 mg/g carbomer gel formulation of FA to dorsal and groin skin from healthy Beagle dogs

The amount of FA applied to canine skin in each diffusion cell ranged from 762 to 1087 μg (mean ± SEM 946 ± 9 μg). All QCs and standards running alongside samples met validation criteria. FA was never detected in any sample from un-dosed control cells, nor detected within quantifiable limits in any receptor fluid sample 24 h after application (Table 1). HPLC-UV analyses indicated that total FA recovery was 90.2 ± 9.0% (range 65–107%) after 24 h; no significant difference was found between skin sites or treatment groups (Table 1). From skin surface swabs, overall recovery of FA was 76.0 ± 17.7% independent of skin site or treatment group. A significantly (*P* = 0.002) higher percentage of the applied dose was found within cryosections from the dorsum (17.7 ± 2.4%) in comparison to the groin (10.7 ± 1.0%; Figure 2).

**Table 1:** Mean (±SEM) individual and combined percentage recoveries of FA

Measurement (%)		Site	Treatment Group		
			Undamaged	Shampooed	Tape-stripped
Site of FA recovery	Total drug	Dorsum	87.2 ± 6.8	93.0 ± 6.7	92.9 ± 3.6
		Groin	89.2 ± 5.4	89.2 ± 7.8	89.9 ± 6.0
	Surface swab	Dorsum	60.4 ± 15.8	80.5 ± 10.0	79.2 ± 6.0
		Groin	77.2 ± 5.9	78.5 ± 12.0	80.4 ± 6.4
	Skin cryosections	Dorsum	26.9 ± 10.2	12.6 ± 4.0	13.7 ± 2.7
		Groin	12.0 ± 3.3	10.7 ± 4.5	9.4 ± 3.6
	Receptor fluid	Dorsum	0.0 ± 0.0	0.0 ± 0.0	0.0 ± 0.0
		Groin	0.0 ± 0.0	0.0 ± 0.0	0.0 ± 0.0

FA: fusidic acid. Mean (±SEM) individual and combined percentage recoveries of FA from in vitro diffusion cells containing dorsal or groin skin from healthy Beagle dogs (*n* = 6) after topical application of a 10 mg/g FA suspension (Isathal®) for 24 h. Skin samples were undamaged, shampooed or tape-stripped (*n* = 6 per group) prior to dosing. After exposure, the skin was swabbed prior to transverse cryosectioning and swabs, skin samples and receptor fluid analysed for FA content

No significant inter-dog variability in FA distribution was seen between skin from the six donor dogs within skin group (undamaged, shampooed or tape-stripped) or site (Additional file 2).

FA was detected in 80% of vials (376 of 470 vials, equivalent to 72.6% of all 518 vials; Table 2) containing cryo-sections where follicular infundibula or more superficial structures (surface hairs, living epidermis, keratin and free hairs) were observed in representative (paired) histological specimens. FA was never detected in the absence of these structures, i.e. in sections containing only isthmi, inferior portions of the hair follicle or subcutaneous fat (*n* = 48 vials; Table 2; *P* < 0.0005, Chi-squared test).

**Table 2:** Comparison between presence of infundibular and more superficial structures and presence of FA

	Vials with FA detected n (%)	Vials without FA n (%)
Infundibulae or surface structures on sections	376 (72.6)	94 (18.1)
No infundibulae or surface structures on sections	0 (0.0)	48 (9.3)

FA: fusidic acid; *P* < 0.0005, Chi-squared test. Topical application of a 10 mg/g FA suspension (Isathal®) for 24 h to dorsal or groin skin from healthy Beagle dogs (*n* = 6) held in diffusion cells; a comparison between the presence of infundibular and more superficial structures (surface hairs, interfollicular epidermis, keratin and free hairs) versus deeper structures (isthmi,inferior portions, subcutaneous fat) on analysis of histological specimens and the presence of FA in the corresponding vials of cryosections (vials, *n* = 518;) when assayed by HPLC-UV

A mean (± SEM) FA concentration of 395.4 ± 30.9 μg/g was found in each treated portion of skin 24 h after topical application (Table 3). No significant differences (*P* > 0.05) were seen between the concentrations achieved in any site or treatment group. The concentration of FA (mean ± SEM) in the uppermost (from epidermal aspect) two vials of cryosections of each skin specimen (approximately 240 μm) was 2000 ± 815 μg/g.

**Table 3:** Mean (± SEM) concentration of FA measured in transverse cryosections

FA concentration (mean μg/g)		Treatment Group		
		Undamaged (*n* = 6)	Shampooed (*n* = 6)	Tape-stripped (*n* = 6)
Site	Dorsum	617.5 ± 135.9	345.0 ± 46.0	346.4 ± 35.4
	Groin	392.3 ± 51.8	298.8 ± 47.9	372.4 ± 69.0

Mean (± SEM) concentration of FA measured in transverse cryosections obtained from full thickness dorsum or groin skin from healthy Beagle dogs (*n* = 6) after topical application of a 10 mg/g FA suspension (Isathal®) for 24 h in four diffusion cell experiments. Skin samples were undamaged, shampooed or tape-stripped (*n* = 6 per group) prior to dosing. After exposure, the skin was swabbed prior to transverse cryosectioning. No significant differences were seen between the concentrations achieved in any site or treatment group (ANOVA, *P* > 0.05)

### Skin measurements

#### Thickness of stratum corneum and whole skin specimens

Dorsal skin thickness (1.1 ± 0.0 cm; range 0.9–1.4 cm) exceeded (*P* < 0.0005) that of groin skin (0.8 ± 0.0 cm; range 0.5–1.1 cm), but overall thickness did not vary between undamaged, tape-stripped or shampooed skin obtained from either site. Stratum corneum thickness showed a similar relationship (dorsum 14.0 ± 2.0 μm; groin 12.0 ± 1.6 μm; *P* < 0.0005). A non-significant reduction (*P* = 0.105) in stratum corneum thickness was seen in the tape-stripped groin skin compared to the undamaged skin (undamaged 12.6 ± 1.9 μm, tape-stripped 11.7 ± 1.1 μm); shampooing had no measureable effect. Sectioning from either dermal or epidermal aspects did not affect stratum corneum thickness measurements (Additional file 3).

#### Hair follicle density

The hair follicle density of dorsal skin (mean ± SEM, 5.8 ± 0.2 compound follicles/mm^2^) exceeded (*P* = 0.004) that of groin skin (2.2 ± 0.6 compound follicles/mm^2^), with a greater percentage of the total sectioned area of skin containing follicular infundibula (dorsum 19.8 ± 1.8%, groin 6.7 ± 1.6%; *P* = 0.004).

#### Electrical resistance

Barrier integrity testing prior to analysis demonstrated that electrical resistance was comparable (*P* = 0.452) between the dorsum and groin skin (dorsum mean ± SEM = 3.71 ± 0.19 kΩ, groin = 3.46 ± 0.16 kΩ).

## Discussion

The combination of transverse histological sectioning and HPLC-UV assessment of FA concentrations in serial sections was pivotal in ascertaining in detail the depth of FA permeation through canine skin. Coupled with the more conventional processes of drug recovery in post-treatment skin, surface swab and receptor fluid samples, these data confirmed FA permeation to the level of the follicular infundibulum and thus, the presence of drug at the level of infection in canine superficial bacterial folliculitis following topical application. Whilst formulation with different vehicles commonly influences skin permeation, we used a commercially available ophthalmological product that contains the same vehicle as a licensed steroid-containing topical skin product for dogs (Isaderm®, DVP) to maximise the clinical relevance of the results of our in vitro study to veterinary practitioners.

This first report of the proportion of FA remaining on canine skin surface after application in vitro (76.0 ± 17.7% at 24 h) was remarkably similar to the 80% figure reported in an analogous study of human skin [12]. The higher hair follicle density dorsally likely accounts for the increased amounts of FA present within skin from this site when compared with groin, particularly since the presence of drug in skin sections was significantly associated with histological observation of infundibulae and other superficial structures.

The failure to detect FA in receptor fluid in this canine study was in accordance with a previous in vitro penetration study of human skin [12], and not un-expected from the physicochemical properties of the molecule [8, 9]. The limit of detection of this HPLC method was well below the predicted concentration of FA in receptor fluid had we reproduced the 1.3% bioavailability described by Degim et al [13]. The full thickness penetration of FA reported in that study might reflect technical or procedural differences such as apparent absence of barrier integrity testing prior to dosing; [13] ensuring that the epidermal barrier layer maintains its integrity is an essential factor to the successful performance of diffusion cell experiments [16]. In this study, electrical resistance was used for barrier integrity testing, but this does not appear to have been described previously for dogs. Values obtained here fell between those reported for rat (3 kΩ) and pig (4 kΩ) skin [19], in parallel with relative stratum corneum thickness in these species (rat 6.0–13.3 μm < dog 9.4–15.1 μm < pig 13.1–18.1 μm) [21]. Comparable electrical resistance and thus barrier integrity in undamaged, shampooed and tape-stripped skin correlated with the equivalent FA penetration across the three groups. Values reported here should be of use for future skin integrity testing for canine in vitro diffusion experiments.

Our model indicates that topical therapy with FA in canine skin is likely to achieve concentrations that markedly exceed MICs of staphylococcal strains deemed both ‘susceptible’ and ‘resistant’ using existing interpretative criteria. By extrapolating the mean FA concentration achieved in the top 240 μm of skin (2000 ± 815 μg/g) using a skin density value of 1.09 [22], the overall concentration of FA in this region can be estimated as 2180 ± 634 mg/l. This markedly exceeds previously reported MIC_90_ of both methicillin-resistant and susceptible *S. aureus* and *S. pseudintermedius* [6, 23], and EUCAST systemic therapy breakpoint for ‘resistance’ (1 mg/l) [24] and compares favourably with MIC_100_ values for FA-resistant MRSA (1024 mg/l) [7]. Development of interpretive criteria for topical rather than just systemic use of antimicrobial therapy is urgently required.

The stratum corneum thickness of undamaged canine skin in this study was closely comparable to those of previous reports [21, 25]. Tape-strip removal of stratum corneum cells and lipid is commonly used to degrade the barrier and enhance drug permeability, although post-stripping measurements of thickness in cryosections (which best preserve stratum corneum architecture in haired skin) [21, 26] are very rarely reported [27–29]. We speculate that the failure of tape stripping to significantly reduce canine interfollicular stratum corneum thickness reflects the combined effects of a dense mat of short stubbly hairs reducing D-squame tape access to the interfollicular epidermis (close clipping was avoided to prevent stratum corneum disruption) [29], uneven skin surface [30], thicker corneum at follicular ostia [31], and or preferential removal of loose corneocytes that may be lost or otherwise not included in measurements of residual compact layers. Further studies that optimise parameters, such as applicator pressures, numbers of repeat strips and clipping methods [20] for the thin but compact corneal layers of canine haired skin, are indicated.

## Conclusions

These data suggest that topical FA should be useful in the treatment of canine surface and superficial pyoderma (intact follicles) caused by bacteria susceptible to fusidic acid, in countries where it is available, but not deep pyoderma (where infection extends to surrounding dermis). Clinical studies are now required to confirm this. Similar studies should now be performed for other topically applied antibiotics to inform evidence-based antibiotic treatment guidelines. Although prevalence of antimicrobial resistance should be monitored prospectively, FA provides an opportunity for topical antibiotic therapy in the treatment of staphylococcal folliculitis in dogs and an option to reduce selection pressure for antimicrobial resistance on these zoonotic canine pathogens from conventional systemic antibiotic use.

## Additional files

Additional file 1: Chemicals used in diffusion cell analysis of fusidic acid permeation into canine skin. (DOCX 15 kb)

Additional file 2: Mean ± SEM percentage of applied dose of fusidic acid recovered. Description of data: Mean ± SEM percentage of applied dose of fusidic acid recovered from swab, within skin or in total for Beagle dogs (*n* = 6) after topical application to undamaged, shampooed or tape stripped dorsum or groin skin. (DOCX 18 kb)

Additional file 3: Mean (± SEM) thickness of stratum corneum measured on vertical cryostat sections. Description of data: Mean (± SEM) thickness of stratum corneum measured on vertical cryostat sections, cut from panniculus up to epidermis or epidermis down to panniculus. Sections taken of full thickness dorsum or groin skin from healthy Beagle dogs (*n* = 6) treated in three ways: undamaged, shampooed or tape-stripped (*n* = 6 per group). (DOCX 15 kb)

## Abbreviations

DVP: Dechra Veterinary Products, Shrewsbury, U.K.
FA: Fusidic acid
HPLC-UV: High pressure liquid chromatography – ultraviolet
LOD: Limit of detection
LOQ: Limit of quantification
MIC: Minimum inhibitory concentration
MRSP: Methicillin-resistant *Staphylococcus pseudintermedius*
OCT: Optimum cutting temperature
QC: Quality control
SEM: Standard error of the mean
