# Supplementary material for: Opportunities for topical antimicrobial therapy: permeation of canine skin by fusidic acid
Source: BMC Vet Res. 2017 Nov 21;13:345. doi: 10.1186/s12917-017-1270-6 (PMC5697365; doi:10.1186/s12917-017-1270-6)
Supplement: Supplementary file 1 — Chemicals used in diffusion cell analysis of fusidic acid permeation into canine skin. (DOCX 15 kb) [file 12917_2017_1270_MOESM1_ESM.docx]

**Chemicals used in diffusion cell analysis of fusidic acid permeation into canine skin.**

| Product | Concentration | Source |
| --- | --- | --- |
| Fusidic acid sodium salt | >98% | Sigma-Aldrich, Irvine, UK |
| Ethanol | >99% laboratory reagent grade | Fisher Scientific, Loughborough, UK |
| Methanol | >99% laboratory reagent grade | Fisher Scientific, Loughborough, UK |
| Glacial acetic acid | >99% laboratory reagent grade | Fisher Scientific, Loughborough, UK |
| Optimal cutting temperature (OCT) compound (Cryo-M-Bed) |  | Bright Instruments, Luton, UK. |
| Fusidic acid eye drop suspension (Isathal^®^) | 10 mg/g | Dechra Veterinary Products Ltd, Shrewsbury, UK. |
| Chlorhexidine and miconazole shampoo (Malaseb^®^) | 2 % of each | Dechra Veterinary Products Ltd, Shrewsbury, UK. |
