# Supplementary material for: Opportunities for topical antimicrobial therapy: permeation of canine skin by fusidic acid
Source: BMC Vet Res. 2017 Nov 21;13:345. doi: 10.1186/s12917-017-1270-6 (PMC5697365; doi:10.1186/s12917-017-1270-6)
Supplement: Supplementary file 2 — Mean ± SEM percentage of applied dose of fusidic acid recovered. Description of data: Mean ± SEM percentage of applied dose of fusidic acid recovered from swab, within skin or in total for Beagle dogs (n = 6) after topical application to undamaged, shampooed or tape stripped dorsum or groin skin. (DOCX 18 kb) [file 12917_2017_1270_MOESM2_ESM.docx]

**Mean ± SEM percentage of applied dose of fusidic acid recovered.**

| Skin Site | Skin treatment | Recovery | Dog | | | | | | Significance of inter-dog variability (*P*<0.05 indicates significance) |
| --- | --- | --- | --- | --- | --- | --- | --- | --- | --- |
|  |  |  | 1 | 2 | 3 | 4 | 5 | 6 |  |
| Dorsum | Undamaged | % recovered on swab | 76.51 ±1.78 | 78.02 ±3.94 | 73.28 ±13.97 | 40.46 ±29.40 | 48.64 ±35.77 | 45.23 ±41.09 | 0.838 |
|  |  | % recovered in skin | 19.45 ±3.30 | 11.56 ±4.31 | 20.51 ±8.71 | 35.18 ±20.48 | 35.94 ±26.07 | 38.70 ±25.38 | 0.838 |
|  |  | Total % recovered | 95.96 ±1.52 | 89.57 ±0.37 | 93.79 ±5.26 | 75.64 ±8.92 | 84.57 ±9.70 | 83.93 ±15.72 | 0.495 |
|  | Shampooed | % recovered on swab | 84.03 ±6.83 | 94.17 ±3.23 | 86.00 ±0.82 | 73.78 ±2.17 | 82.03 ±3.21 | 62.70 ±0.08 | 0.087 |
|  |  | % recovered in skin | 12.09 ±4.13 | 10.45 ±0.91 | 7.20 ±0.45 | 14.25 ±1.78 | 11.09 ±4.48 | 20.22 ±1.98 | 0.189 |
|  |  | Total % recovered | 96.12 ±2.70 | 104.62 ±2.32 | 93.20 ±1.27 | 88.03 ±0.40 | 93.12 ±1.27 | 82.92 ±2.06 | 0.080 |
|  | Tape stripped | % recovered on swab | 73.29 ±4.44 | 80.25 ±3.44 | 88.85 ±0.51 | 74.31 ±9.36 | 84.75 ±1.11 | 73.74 ±8.65 | 0.221 |
|  |  | % recovered in skin | 17.71 ±2.36 | 11.74 ±3.11 | 9.39 ±4.68 | 15.60 ±4.05 | 12.58 ±0.76 | 14.97 ±3.25 | 0.549 |
|  |  | Total % recovered | 91.00 ±2.10 | 91.98 ±0.33 | 98.24 ±4.17 | 89.91 ±5.32 | 97.33 ±1.87 | 88.70 ±5.40 | 0.321 |
| Groin | Undamaged | % recovered on swab | 73.44 ±17.48 | 68.16 ±2.76 | 74.19 ±5.13 | 78.91 ±5.78 | 82.95 ±13.56 | 85.30 ±8.26 | 0.676 |
|  |  | % recovered in skin | 8.55 ±0.59 | 17.66 ±1.16 | 10.24 ±2.21 | 14.53 ±5.23 | 8.86 ±4.25 | 12.04 ±3.18 | 0.329 |
|  |  | Total % recovered | 81.98 ±16.89 | 85.82 ±1.60 | 84.51 ±3.01 | 93.43 ±0.55 | 91.81 ±9.32 | 97.34 ±5.08 | 0.583 |
|  | Shampooed | % recovered on swab | 79.77 ±0.80 | 57.16 ±21.39 | 68.93 ±3.13 | 85.53 ±6.94 | 91.67 ±2.27 | 88.19 ±1.91 | 0.122 |
|  |  | % recovered in skin | 9.32 ±1.29 | 19.84 ±13.13 | 12.17 ±1.13 | 9.08 ±5.84 | 7.56 ±0.29 | 6.00 ±0.30 | 0.475 |
|  |  | Total % recovered | 89.09 ±2.09 | 77.01 ±8.26 | 81.10 ±4.26 | 94.61 ±1.10 | 99.22 ±2.55 | 94.18 ±1.61 | 0.069 |
|  | Tape stripped | % recovered on swab | 71.14 ±12.08 | 81.44 ±1.66 | 73.41 ±7.20 | 80.92 ±11.94 | 88.77 ±1.96 | 86.98 ±1.63 | 0.337 |
|  |  | % recovered in skin | 9.06 ±0.42 | 14.67 ±7.22 | 13.58 ±5.59 | 4.83 ±1.58 | 6.37 ±0.47 | 8.11 ±2.56 | 0.306 |
|  |  | Total % recovered | 80.20 ±11.67 | 96.12 ±8.86 | 86.99 ±1.61 | 85.75 ±10.36 | 95.13 ±1.49 | 95.09 ±0.93 | 0.506 |

*Mean ± SEM percentage of applied dose of fusidic acid recovered from swab, within skin or in total for Beagle dogs (n=6) after topical application to undamaged, shampooed or tape stripped dorsum or groin skin.*
