# Supplementary material for: Opportunities for topical antimicrobial therapy: permeation of canine skin by fusidic acid
Source: BMC Vet Res. 2017 Nov 21;13:345. doi: 10.1186/s12917-017-1270-6 (PMC5697365; doi:10.1186/s12917-017-1270-6)
Supplement: Supplementary file 3 — Mean (± SEM) thickness of stratum corneum measured on vertical cryostat sections. Description of data: Mean (± SEM) thickness of stratum corneum measured on vertical cryostat sections, cut from panniculus up to epidermis or epidermis down to panniculus. Sections taken of full thickness dorsum or groin skin from healthy Beagle dogs (n = 6) treated in three ways: undamaged, shampooed or tape-stripped (n = 6 per group). (DOCX 15 kb) [file 12917_2017_1270_MOESM3_ESM.docx]

**Mean (± SEM) thickness of stratum corneum measured on vertical cryostat sections**

| Site | Sectioning direction | Treatment group | | |
| --- | --- | --- | --- | --- |
|  |  | Undamaged | Shampooed | Tape-stripped |
| Dorsum | Epidermis > Panniculus | 14.50±1.28 | 12.80±2.00 | 14.69±1.65 |
|  | Panniculus > Epidermis | 15.90±96 | 13.29±1.31 | 14.34±2.51 |
| Groin | Epidermis > Panniculus | 12.58±2.27 | 11.77±1.08 | 11.43±0.72 |
|  | Panniculus > Epidermis | 12.56±1.37 | 12.48±1.82 | 11.98±1.20 |

*Mean (± SEM) thickness of stratum corneum measured on vertical cryostat sections, cut from panniculus up to epidermis or epidermis down to panniculus. Sections taken of full thickness dorsum or groin skin from healthy Beagle dogs (n=6) treated in three ways: undamaged, shampooed or tape-stripped (n=6 per group).*
